# 

**Published:** 1893-05-06

**Authors:** 


**OUR NEW OFFICES.**

428, Strand, W.C., will henceforth be the Office of this Journal.

**Notices of Births, Deaths, and Marriages,** are inserted in this column at a charge of 2s. 6d. for an announcement not exceeding four lines.

### NOTICE TO CORRESPONDENTS.

All MS., Letters, Books for Review, and other matters intended for the Editor should be addressed The Editor, The Lodge, Porchester Square, London, W.

The Editor cannot undertake to return rejected M.S., even when accompanied by stamped directed envelope.

**Notice to Advertisers and Subscribers.**

All Advertisements, Orders for Copies of The Hospital, and Business Communications should be addressed to The Publisher, *not to the Editor*, The Hospital, 428, Strand, W.C.

The Cost of Subscription, post free, is as follows:—Per week, 2½d.; per quarter, 2s. 6d.; per half-year, 5s.; per annum, 8s. 6d. Foreign:—Per annum, 10s. 8d.; payable, in all cases, in advance.

**"Burdett's Hospital Annual," 1893.**

Fourth year of publication. 700 pp. Boards, gilt lettering. A most complete guide to British, Colonial, Indian, and American Hospitals, Dispensaries, Nursing and Convalescent Institutions. and Asylums. Price 5s. Published by The Scientific Press (Limited), 428, Strand, W.C.

**NOTICE.—The Index for Vol. XII. (ending September 1892) is on sale at the Office of this Journal, price 2½d., post free.**

### Books Received.

Elliot Stock.
"How to Improve the Physique." By Medicus.

The Engineering Record Publishing Co.

"Ventilation and Heating." By John S. Billings, M.D.

David Stott.

"Voluntaries for an East London Hospital." By the Earl of Lytton, the Bishop of Bedford, and others.

Percival and Co., London.
"Introduction to the Antiseptic Treatment of Wounds According to the Method in Use at Professor Billroth's Clinic, Vienna." By Dr. Victor V. Hacker.

MacMillan and Bowes.

"Some Lectures." By Sir George Paget, M.D. Edited by Charles E. Paget.

Swan Sonnenschein and Co.
"Drunkenness." By George R. Wilson, M.B.
"Margate as a Health Resort." By H. Evelyn Crook, M.D.

### SCRAPS AND GLEANINGS.

There are fewer blind persons in the United States than any other country.

The Goldsmiths' Company have contributed £5,000 to the fourth quinquennial Maintenance Fund of the London Hospital.

Even as lately as 1883 the National Debt stood at 713 millions; it is now 579½ millions. This year it is six millions less than last year.

Mr. Eddison, of phonograph fame, is a substantial supporter of female work, no less than 200 employés finding means of livelihood in his machinery works.

Miss Martha Wilson gave the land and 9,000 dollars for the erection of a new hospital at Mount Vernon, New York, the foundation-stone of which has recently been laid.

A "monopolist," after all, is a term requiring definition, each country having its own interpretation thereof. In Japan a monopolist is any farmer who owns ten acres.

A recent invention of an ingenious clockmaker, is an instrument which will go ten years without winding; how long it takes to actually complete the winding we are not told.

The General Hospital at Jersey report a favourable balance at their annual meeting, the funds being £2,244 to the good. There were 318 inmates of the hospital in December last.

It is said that cholera is spreading all over Central Russia; the peasants are dying by thousands, and no reports are allowed to be published concerning the ravages of this epidemic.

At a meeting of the Canterbury Town Council the Infectious Diseases Hospital Committee report that it is finally decided to erect an Infectious Disease Hospital in that locality.

Dr. Matias Nielo Serrano, editor of the leading Spanish medical paper, has had the title of Marquis of Quadalerzas conferred upon him in recognition of the services he has rendered to his country through science.

A reform long needed in America has seen its accomplishment in the passing of the State Bill, levying a tax to carry out the law providing for the insane. The annual income to be derived from this tax will be about 1,350,000 dollars.

Owing to the immense increase in the number of cases of "glanders" the London County Council are endeavouring to check the spread of the disease by authorising the adoption of drastic measures on the part of the veterinary authorities.

It is proposed to erect an isolation and a children's ward to the Taunton and Somerset Hospital as a memorial to the late eminent surgeon and philanthropist, Mr. W. Liddon, who devoted thirty years of self-sacrificing labour to the patients of that hospital. The sum required is £5,000.

The annual festival of the North Eastern Hospital takes place on the 9th May. Last year there were 556 in-patients in the Hospital, and the out-patient department had 315,123 new cases, with the large total of 1,108,816 attendances. The sum of £20,000 is still needed to complete the enlargement fund.

At the annual meeting of the Committee of the Scarborough Hospital and Dispensary, the Mayor in adopting the report, remarked that it would be the last one emanating from the Old Elder Street Hospital. The balance-sheet showed the income to have been £1,210 as against £1,010 for the previous year.

A dispensary, conducted entirely by female physicians, is to be found at Baltimore, a city which, after the completion of the new medical school where women and men are to be admitted on equal terms, will have no rival in the world in its encouragement of scientific pursuits for both sexes.

The cargo of the "Hilda" just arrived in the Mersey from Montserrat, West Indies, consists almost entirely of lime juice, of which she brings no less than 50,000 gallons. This vessel is now regularly engaged in bringing over lime juice, which is consigned solely to one firm, Messrs. Evans and Co., of Liverpool.

The efficiency of the Worcester General Infirmary is well maintained; the number of in-patients received during the year was 1,219. The committee at the general meeting referred to the benefits the Hospital had received through the generosity of the late Mr. Ganderton, who at various times had contributed money to the extent of £12,000.

The Board of the General Hospital, Cheltenham, view with very great concern the very serious decrease of annual subscriptions during the past three years. The total receipts for the past year (exclusive of balances and amounts drawn from the legacy fund) was £4,049. The ordinary expenditure being £4,901, leaving £851 in excess of receipts.

At the annual meeting of the governors of the Leicester Infirmary the question of the direct representation of the working classes on the weekly board was discussed. In virtue of the liberality with which the artisans support the institution, it was admitted that they had the right of being represented, and a representative was accordingly chosen.

The 80th annual dinner of the London Orphan Asylum was held on the 19th ult. at the Hotel Metropole. The charity was founded in 1813 for the benefit of orphans of those in a good position of life. The number of inmates has risen from 156 to 513. The institution is well managed, and worthy of support. Donations of £2,957 were announced during dinner.

The South Infirmary at Cork reports at its annual meeting that the number of patients admitted during the year amounted to 922, the out-patient department numbering 6,721. The receipts for the year 1892 amounted to £3,254. The liabilities of the institution were rendered heavy by necessary changes made in the laundry, and a balance is still due to the bankers.

The quarterly report of the Devonshire Hospital and Buxton Bath Charity is always worth reading, the general condition of the institution being uniformly satisfactory, and its financial one is good also. There has been an increase in the amount of the casual as well as of the annual subscribers, and the result is that the balance in hand at the end of the year amounted to £387.

### FIRST IMPRESSIONS OF BOOKS RECEIVED.

[It is requested that all books intended to be noticed in this column may be sent to the Editor, at the Office, 428, Strand, W.C., not later than Tuesday evening in each week.]

The Medical Pioneer, April, 1893.

This is the organ of the British Medical Temperance Association, and it deals with the medical aspect of the crusade against drunkenness. This number contains a good portrait and short biography of Dr. Morton, of Kilburn, and among other papers one by Dr. Ridge on substitutes for "Alcohol," and an account of the annual meeting of the London Temperance Hospital.

Aids to Biology. By Joseph W. Williams. (London: Bailliere, Tindall, and Cox.)

This little volume is specialy prepared to meet the requirements of students reading for the first examination of the Conjoint Board of England, and its scope is limited by the syllabus of that examination. Mr. Williams has previously written elementary science manuals, and he can express himself tersely and clearly. The little book before us is very simple in arrangement, accurate in statements, and well designed to meet the author's purpose.

Notes upon an Outbreak of an Unusual Form of Skin Disease at the Greenock Parochial Asylum and Poorhouse. By Frank Ashby Elkins, M.B., Senior Assistant Physician, Royal Edinburgh Asylum. (London: J. W. Danks and Co.).

This is a reprint of a paper which originally appeared in "The Medical Press and Circular." It records an epidemic of a skin disease which occurred in the autumn of 1888, and resembled in many of its features that which prevailed in some of the London Workhouses in 1891. The disease was characterised by marked constitutional depression and a general papular rash which often becomes vesicular or even pustular. The rash in some cases resembled that of

